# Investigation of lightning ignition characteristics based on an impulse current generator

**DOI:** 10.1002/ece3.5855

**Published:** 2019-12-02

**Authors:** Junwei Feng, Hao Shen, Dong Liang

**Affiliations:** ^1^ Safety Engineering Research Center School of Intelligent System Engineering Sun Yat‐sen University Guangzhou China; ^2^ Guangdong Provincial Key Laboratory of Fire Science and Technology Guangzhou China

**Keywords:** conifer needle, impulse current, lightning fire, prediction model

## Abstract

Lightning strike is an important ignition source of forest fires. Artificial lightning discharge is a method for studying lightning fires. However, there is not enough data on the ignition of combustible materials caused by artificial lightning discharge. Previous studies on lightning ignition have focused on the heating and ignition effects of long continuing current (LCC), but the function of the impulse current that occurs before the LCC has not been taken into account. In this paper, an impulse current generator of 8/20 μs was used to simulate the ignition effect of impulse current on conifer needle beds. Different current waveforms have different ignition characteristics. We compared five kinds of conifer needle beds. The average of the current needed to ignite the needle bed of *Larix gmelinii *(Ruprecht) Kuzeneva was the smallest, and the average of the breakdown voltage was the smallest for the needle bed of *Pinus massoniana* Lamb. The total energy input to the conifer needle beds was fitted as a multiple log‐linear regression model. The heating energy proportion value varies with different bulk densities, current amplitudes, and moisture contents. Based on this data, the heating energy of the impulse current transferred to the needles can be predicted. This information in conjunction with previous research on LCC was used to derive a lightning ignition prediction model of the full waveform for conifer needle beds.

## Graphical abstract

With the rise of global temperature, the forest lightning fire is very serious. Forest fires have seriously damage to the ecosystem and cause great economic losses. In this manuscript, the ignition characteristics of lightning strike were studied, and the prediction model of lightning ignition was established.

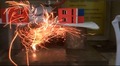

## 1 INTRODUCTION

Lightning strike is an important ignition source of forest fires, resulting in great losses and harm. The mechanism of ignition is complex. The Da Hinggan Mountains and Hulunbuir are the main areas that experience lightning fires in China. The Da Hinggan Mountains exhibit the highest concentration of lightning fires and experience these fires almost every year. Lightning fires accounted for 38% of the total forest fires in the Da Hinggan Mountains and 18% in Hulunbuir (Shu, Wang, Tian, Li, & Xiao, 2003; Tian et al., 2011; Tian, Shu, Wang, & Zhao, 2009). On June 2, 2018, lightning fires occurred in the Da Hinggan Mountains of Inner Mongolia. A total of 4,300 people gathered to fight the fires for nearly a week (Liu & Zhang, 2018). Therefore, it is important to study lightning fires in China.

According to the International Electrotechnical Commission (IEC) and the Society of Automotive Engineers (SAE) standards, the typical definition of lightning current is shown in Table 1 (IEC 1024‐1, 1990; SAE ARP 5412 2005). The maximum value of impulse current (part A) ranges up to 200 kA, but the duration is <500 μs. The long continuing current (part C) is within the range of 200–800 A, but it lasts for 1 s. It is generally believed that the main cause of forest fires is the existence of long continuing current (LCC) in the lightning waveform (Fuquay, Taylor, Hawe, & Schmid, 1972). However, the impulse current energy accounts for a large portion of the energy of lightning (Dong, 2015; SAE ARP 5412, 2005). In fact, the impulse current can ignite some flammable matter.

**Table 1:** The typical definition of lightning current

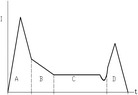	Definition
First return stroke (part A)	The maximum value of current is 200 kA. The rise time is <50 µs. The current attenuates to 1% of the peak is no more than 500 µs
Intermediate current (part B)	The average value of current is 2 kA. The duration is <5 ms
Long continuing current (part C)	The value of current is 200–800 A. The duration is 0.25–1 s
Subsequent stroke (part D)	The maximum value of current is 100 kA. The rise time is <25 µs. The current attenuates to 1% of the peak is no more than 500 µs

Note

The typical lightning current waveform is composed of four parts: first return stroke, intermediate current, long continuing current, and subsequent stroke according to the International Electrotechnical Commission (IEC) and the Society of Automotive Engineers (SAE) standards.

Taylor proposed a hypothesis about the mechanism of lightning fires: A lightning discharge striking and rupturing a live conifer produces and ignites a mixture of volatile extractives and finely divided bark, wood, and needle particles to an intense, short‐lived ball or column of fire, which in turn ignites flash fuels in the tree crown or on the forest floor (Li & Hu, 2004; Taylor, 1973). Fuquay, Aughman, and Latham (1979), Anderson (1993), and Sun, Yao, Han, and Chen (2006) compared the energy produced by lightning and the energy required for combustible ignition, explored the mechanism of lightning ignition, and then obtained the theoretical discrimination formula of lightning ignition. However, the ignition discrimination formula is mainly used in the prediction of LCC ignition, and the effect of impulse current is not considered.

At present, the empirical model of lightning ignition can be explored by artificial lightning generators. The common empirical model for lightning ignition is logistic regression analysis. It is a nonlinear method of binary dependent variable regression analysis. Logistic models account for the many variables used in the experiment, and the relationship between ignition probability and various influencing factors can be expressed as a logistic regression equation (Zhu, Liu, Deng, & Zhang, 2012). Latham and Schlieter (1989) and Zhu et al. (2012) derived the logistic regression equation of LCC ignition through a large number of artificial lightning experiments. This equation was then used to predict the probability of lightning fires. Darveniza and Zhou (1994) studied the influence of the impulse current on combustible ignition using an impulse current generator. They found that the impulse current has a great mechanical effect in addition to its heating effect, and it can destroy the combustible material.

In recent years, most research on the forest lightning fire is based on statistical analysis of historical data. These studies identified the spatial and temporal distribution of lightning fire in various regions. Wierzchowski, Heathcott, and Flannigan (2002) studied the pattern of lightning fires on the east and west sides of the Canadian mountain boundary. There was an average of one fire for every 50 lightning discharges in British Columbia, whereas there was one fire for every 1,400 lightning discharges in Alberta. Wotton and Martell (2005) studied the Ontario region and found that moisture content was the main factor controlling the undergrowth lightning fire, and the positive cloud‐to‐ground lightning strikes were more dangerous than the negative lightning strikes. Lutz, Wagtendonk, Thode, Miller, and Franklin (2009) studied Yosemite National Park and speculated that the reductions in the snow fields will increase forest lightning fires as the global temperature continues to rise. A pair of studies (Anderson, 2002; Anderson, Martell, Flannigan, & Wang, 2000) put forward a model to predict the probability that a lightning flash will lead to a detectable fire. This is done by estimating the probability of a long continuing current in the lightning strike, the probability of ignition, the probability of survival, and the probability of arrival. Nieto, Aguado, García, and Chuvieco (2012) established a logistic model for predicting lightning fire in Madrid and Aragon, Spain. After variable selection, the number of thunderstorms was the most significant factor in the model. Using the atmospheric stability index and fuel moisture code as independent variables, Magnussen and Taylor (2012) predicted the daily lightning fire risk in British Columbia, Canada with a logistic model.

In summary, previous studies of heating by impulse current have primarily focused on qualitative descriptions, and these studies have lacked quantitative research. In this paper, we analyzed the ignition characteristics of conifer needle beds from the perspective of the energy balance by using an artificial impulse current generator. An empirical prediction equation was obtained via fitting experimental data.

## 2 METHODS

Lightning strikes involve two steps. The first step is the breakdown of combustibles via high voltage. The second step is the current flow that heats the combustibles and initiates ignition. In this paper, the breakdown voltage of different needle beds was first compared. Second, we carried out the ignition characteristics of needle beds and obtained a prediction model of lightning ignition.

### 2.1 Materials

**Figure 1:**
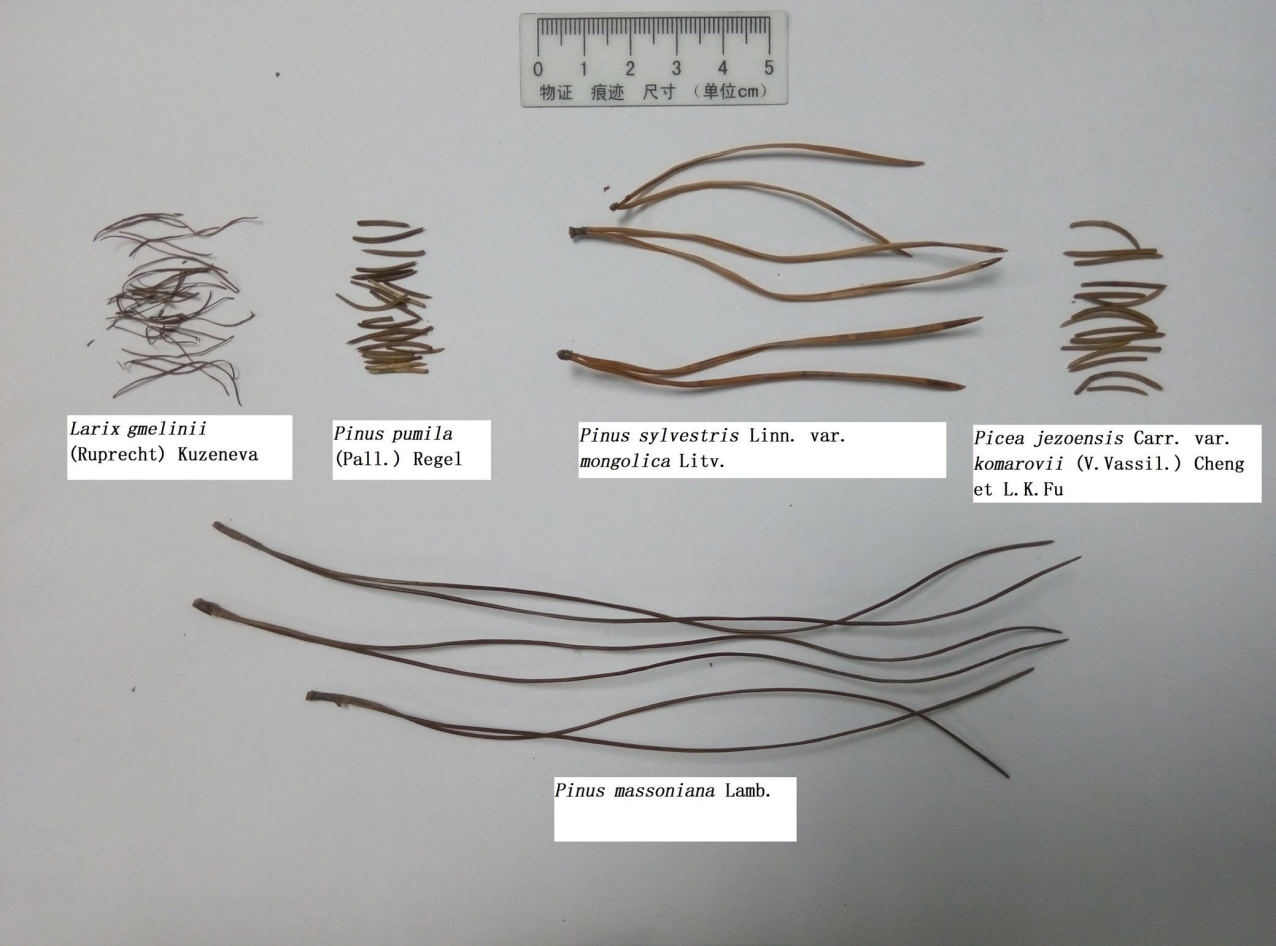
Needles of *Larix gmelinii *(Ruprecht) Kuzeneva, *Pinus pumila* (Pall.) Regel, *Pinus sylvestris* Linn. var. mongolica Litv., *Pinus massoniana* Lamb., and *Picea jezoensis* Carr. var. komarovii (V.Vassil.) Cheng et L.K.Fu used in the experiments. They were collected from the forest floor and dried by the oven. The fuel bed before the experiment is shown in Figure S1

**Figure 2:**
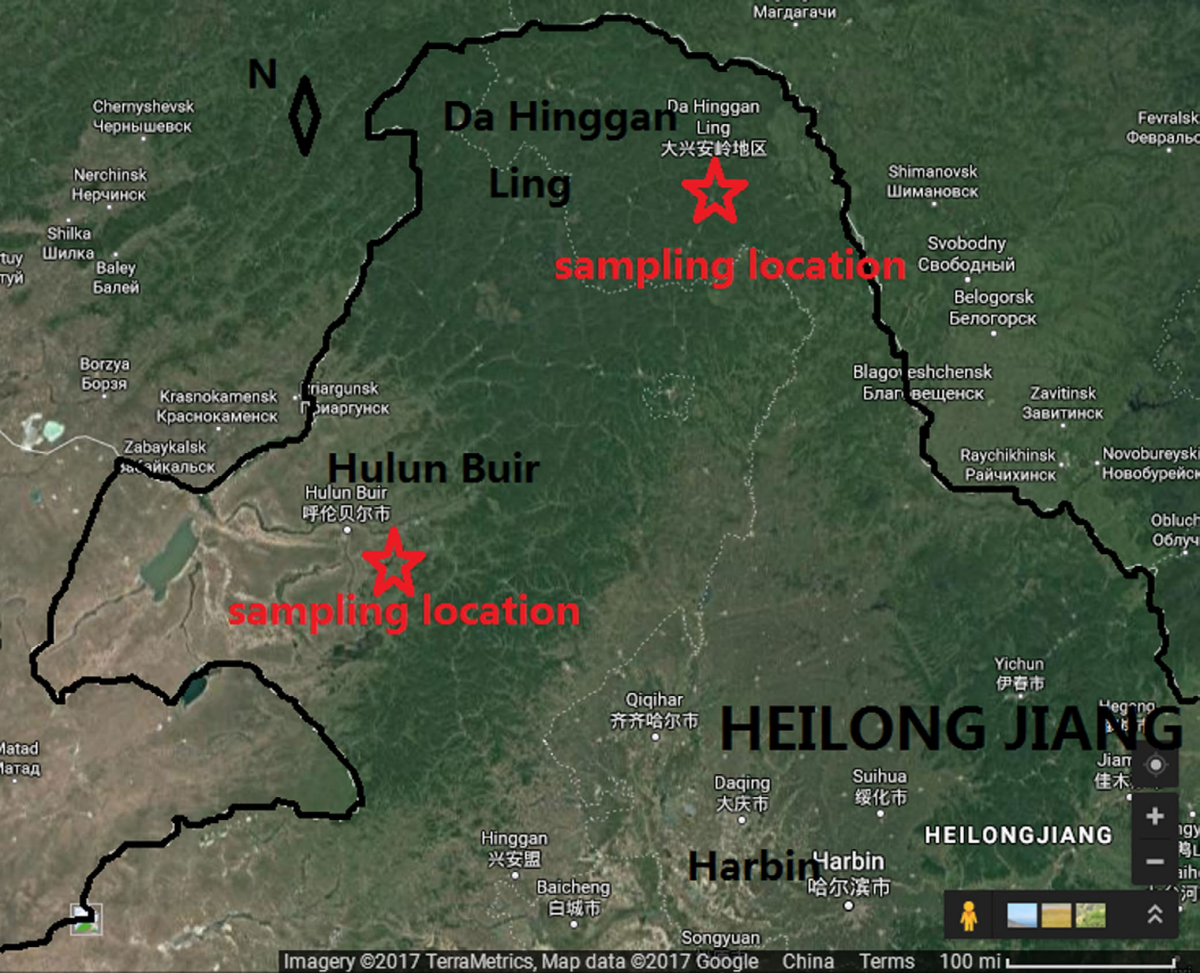
The sampling locations. The geographic information was obtained from Google Maps

The experiment materials were needles of *Larix gmelinii* (Ruprecht) Kuzeneva, *Pinus pumila* (Pall.) Regel, *Pinus sylvestris *Linn. var. mongolica Litv., *Pinus massoniana* Lamb., and *Picea jezoensis* Carr. var. komarovii (V.Vassil.) Cheng et L.K.Fu, as shown in Figure 1. The properties of conifer needles are shown in Table 2. The conifer needles were collected from the forest floor of the New Forest area in the Da Hinggan Mountains (latitude 51°39′45″, longitude 124°23′19″) and Hulunbuir Hailar district (latitude 49°12′41″, longitude 119°43′26″). They are located in the monsoon climate of medium latitudes. These are the main areas for lightning fire in China. The sampling locations are shown in Figure 2.

**Table 2:** Properties of conifer needles

	Needles of *Pinus sylvestris *Linn. var. mongolica Litv.	Needles of *Pinus massoniana *Lamb.	Needles of *Pinus pumila *(Pall.) Regel	Needles of *Picea jezoensis *Carr. var. komarovii (V.Vassil.) Cheng et L.K.Fu	Needles of *Larix gmelinii *(Ruprecht) Kuzeneva
Diameter (mm)	0.99 ± 0.07	0.92 ± 0.09	0.88 ± 0.07	1.05 ± 0.13	0.42 ± 0.09
Length (cm)	7.73 ± 0.64	18.36 ± 2.35	1.48 ± 0.18	1.82 ± 0.21	2.22 ± 0.35
Density (g/cm^3^)	0.510 ± 0.083	0.483 ± 0.105	0.315 ± 0.032	0.348 ± 0.021	0.350 ± 0.045

Note

Each kind of conifer needle was repeatedly measured 10 times, and the average value and *SD* were taken. The moisture content of conifer needle was 0%.

With reference to Wu (2014) and Liu, Wu, and Xie (2014), the preparation methods of samples with different bulk densities were as follows: the samples were laid layer by layer in a fixed volume discharge box. When the thickness of the samples reached the approved height, we stopped loading samples, measured the mass of the samples in the discharge box, and calculated the bulk density of the fuel bed. The preparation methods of samples with different moisture contents were as follows. We obtained the dry samples after drying in the oven. Next, we sprayed the dry samples carefully and evenly. The wet samples were then placed in the constant temperature and humidity chamber so that the samples could fully absorb the moisture. Finally, the samples were weighed before the experiment, and the moisture contents of the samples were obtained. In order to avoid a nonuniform influence of fuel bed bulk density and moisture content, each group of experiments was repeated to obtain the average value and *SD*.

### 2.2 Device

**Figure 3:**
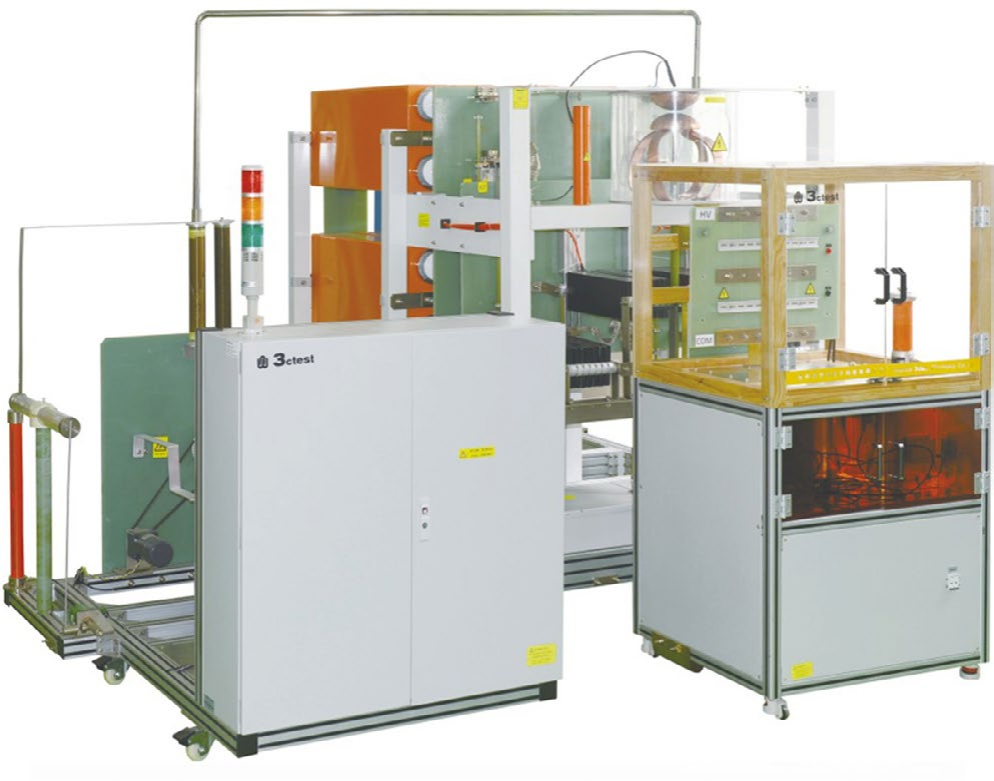
The impulse current generator. It was produced by the Suzhou Test Company, China. This equipment is available at http://www.3ctest.cn/product/show/675. The product information is shown in Table S1

**Figure 4:**
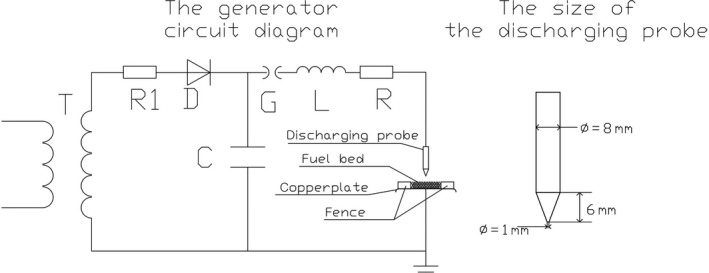
The generator circuit diagram. The diameter of the discharging electrode was 8 mm

The impulse current generator used in the experiments was the automatic impulse current test system LCG 120C (made by TEST Suzhou), as shown in Figure 3. It can generate an 8/20 μs impulse current waveform. The maximum current ranges up to 120 kA. When the sample is loaded in the system, the circuit parameters of the system will change because of the resistance and inductance of the sample, and the actual output waveform will change slightly. The generator circuit diagram is shown in Figure 4. The capacitor is charged and then discharges into the needle bed. The charging circuit consists of the high voltage test transformer (T), charging resistor (R1), storage capacitor (C), and diode (D). The discharge circuit consists of the storage capacitor (C), triggering gap (G), inductance (L), resistor (R), and needle bed. The current through the needle bed is measured by a Rogowski coil. The voltage is measured by a voltage divider. The voltage applied across the needle bed is equal to the voltage applied across the needle bed + air gap minus the voltage of the air gap under roughly the same current. The direction of the current between the discharging electrode and the copper plate can be changed via polarity conversion.

Some studies have also used the impulse current generator to study the lightning damage effect on carbon fiber (Dong, Guo, & Sun, 2015; Guo, 2014; Liu, Yue, Wang, & Ji, 2015). The distance between the tip of the discharging electrode and the surface of the specimen was 2.5 mm in the study of Dong et al. (2015), and the diameter of the discharging electrode was 8 mm. In this article, the distance between the tip of the discharging electrode and the top surface of the needle bed was 2 mm. The size of the discharging electrode is shown in Figure 4.

**Figure 5:**
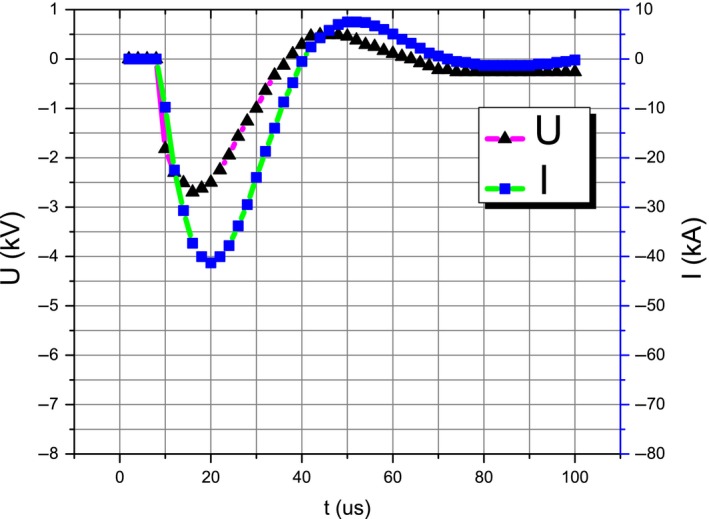
The typical voltage and current curves. *I*_p_ was 41.30 kA, *ρ* was 0.064 g/cm^3^, *x* = 10.7%, and *h* = 3 mm. Needle bed of *Larix gmelinii *(Ruprecht) Kuzeneva. The waveform was 8/20 µs. The current direction was plate‐to‐electrode

The impulse current waveform can be defined by the front time (*t*_f_), the half‐wave time (*t*_h_), and the maximum value (*I*_p_). For example, 8/20 μs indicates that the front time (*t*_f_) is 8 μs and the half‐wave time (*t*_h_) is 20 μs. The typical voltage and current curves are shown in Figure 5.

We used an impulse voltage generator to compare the breakdown voltage values for beds of different conifer needles. The impulse voltage generator can generate an impulse voltage waveform of 1.2/50 μs. This waveform is commonly used in the literature (Deng, He, & Ma, 2011; Lv, Zhou, & Li, 2014).

### 2.3 The experimental process of different conifer needle beds

To compare different conifer needle beds ignited by lightning strike, we tested the *I*_p_, $\int I^{2}dt$, and ∫UIdt of critical ignition for five kinds of conifer needle beds. *I*_p_ is the maximum value of impulse current waveform. $\int I^{2}dt$ is the time integral for square of current curve. ∫UIdt is the total energy of lightning. Keeping the 8/20 µs waveform, we increased the current until it ignited the needle beds and repeated the experiment five times to obtain the average value and *SD*. We did not test for statistical significance because of the small sample size. The parameters of needle beds were 3 mm thickness, 0% moisture content, and 0.1592 g/cm^3^ bulk density. The current direction was plate‐to‐electrode. The porosity of *P. sylvestris* Linn. var. mongolica Litv., *P. massoniana* Lamb., *P. pumila* (Pall.) Regel, *P. jezoensis* Carr. var. komarovii (V.Vassil.) Cheng et L.K.Fu, and *L. gmelinii *(Ruprecht) Kuzeneva needle beds were 68.8%, 67.0%, 49.5%, 54.3%, and 54.5%, respectively.

To compare the breakdown voltage of different conifer needle beds, we increased the peak voltage of the waveform (*U*_p_) incrementally until the needle bed was broken down. Each voltage was repeated five times. A voltage was recorded as the breakdown voltage value if it was able to break down the needle bed in each of the five trials. Using the method of Lv et al. (2014) and Zhou (2015), we repeated this process six times and obtained six breakdown voltage values. Then, we obtained the average value of the six replicates and the *SD*. We did not test for statistical significance because of the small sample size. The parameters of needle beds were 1 mm thickness, 0% moisture content, and 0.0955 g/cm^3^ bulk density. The porosity of *P. sylvestris* Linn. var. mongolica Litv., *P. jezoensis* Carr. var. komarovii (V.Vassil.) Cheng et L.K.Fu, *P. massoniana* Lamb., *P. pumila* (Pall.) Regel, and *L. gmelinii *(Ruprecht) Kuzeneva needles beds were 81.3%, 72.6%, 80.2%, 69.7%, and 72.7%, respectively.

### 2.4 The experimental process of different current waveforms

The IEC standard has defined various impulse current waveforms. Different waveforms are suitable for different situations. The two most widely used waveforms are 8/20 and 10/350 μs. The ignition characteristics of the same fuel under the two waveforms are worthy of our attention. In addition, the direction of the current between the discharging electrode and the copper plate also has an effect on the ignition.

To compare ignition under different impulse current waveforms, we maintained the 8/20 µs or 10/350 µs waveform and increased the current until it ignited the needle beds. We repeated the experiment five times to obtain the average value and *SD*. We did not test for statistical significance because of the small sample size. The parameters of *L. gmelinii *(Ruprecht) Kuzeneva needle beds were 10.7% moisture content, 3 mm thickness, and 0.1062 g/cm^3^ bulk density.

### 2.5 The experimental process of the prediction model of lightning ignition

The lightning ignition theory from Anderson (1993) is shown in Equations (1), (2), (3):

(1) $$E_{c}\geq E_{\text{ig}}$$

(2) $$E_{c}=\int UIdt$$

(3) $$E_{\text{ig}}=\rho \pi r^{2}h\left\{c_{p}\left(T_{\text{ig}}-T_{0}\right)+x\left[c_{\text{pl}}\left(T_{s}-T_{0}\right)+h_{\mathrm{lg}}\right]\right\}$$

*E*_c_—total energy of lightning, which contains the energy of impulse current (*E*_c,impulse_) and the energy of LCC (*E*_c,LCC_), J; *E*_ig_—ignition energy of combustible, J; *U*—voltage drop of lightning channel, V; *I*—current of lightning channel, A; *ρ*—bulk density of needles bed, kg/m^3^; *r*—radius of lightning channel, m; *h*—combustible thickness of needles bed, m; *c*_p_—specific heat of combustible, J/(kg °C); *T*_ig_—ignition temperature of combustible, °C; *T*_0_—ambient temperature, °C; *T*_s_—boiling point of water at atmospheric, °C; *x*—moisture content; *c*_pl_—specific heat of water, J/(kg °C); *h*_lg_—latent heat of water, J/kg.

There are some problems in the practical application of this formula. (a) It is difficult to monitor the voltage drop (*U*) when the lightning is passed through the fuel bed, and this makes it difficult to apply the equation. (2) The total energy of lightning can be described as ∫UIdt, but the energy used for heating and ignition is less than this value. This is because a large portion of the total energy is dissipated by mechanical work and heating up the surrounding air. Therefore, it is a heating efficiency, *φ*, which is used to evaluate the proportion of heating energy to the total energy (∫UIdt). Moreover, the voltage drop across the lightning channel of a combustible material is associated with the current and the properties of the combustible material. Therefore, we can convert the *U* value into *I*, *ρ*, and *x*.

Shu et al. (2003) reported that *L. gmelinii *(Ruprecht) Kuzeneva is the dominant tree and has a wide distribution in area of the Da Hinggan Mountains prone to lightning fires. Therefore, we will build a prediction model of the needle beds of *L. gmelinii *(Ruprecht) Kuzeneva under the 8/20 µs waveform with the current direction of plate‐to‐electrode.

We measured the relationships of the *E*_c,impulse_ to *I*_p_, *ρ*, and *x* by keeping the needle beds at a thickness of 3 mm while varying *I*_p_, *ρ*, and *x*. We set five levels for each independent variable. *I*_p_ was set as 20, 50, 60, 80, and 100 kA. *ρ* was set as 0.0318, 0.0637, 0.1062, 0.1274, and 0.1699 g/cm^3^. *x* was set as 0%, 10.7%, 40%, 80%, and 120%. In nature, most of the impulse currents are in this range. The range of bulk density is from sparse to dense, which is suitable for most natural needle beds. We combined the three independent variables to obtain 5 × 5 × 5 = 125 groups of needle bed types. Each group had different *I*_p_, *ρ*, and *x* values.

Referring to the research of Fuquay et al. (1979), the radius of the lightning channel was taken as 1 cm. In addition, the specific heat and ignition temperature of needles were taken as 2 kJ/(kg °C) and 400°C. We calculated the *E*_ig_ and then calculated the heating efficiency (*φ*) of the total energy of lightning strikes using the critical data of ignition and nonignition (*φ* = *E*_ig_/*E*_c,impulse_ in critical data of ignition and nonignition).

## 3 RESULTS

### 3.1 Experimental phenomenon

**Figure 6:**
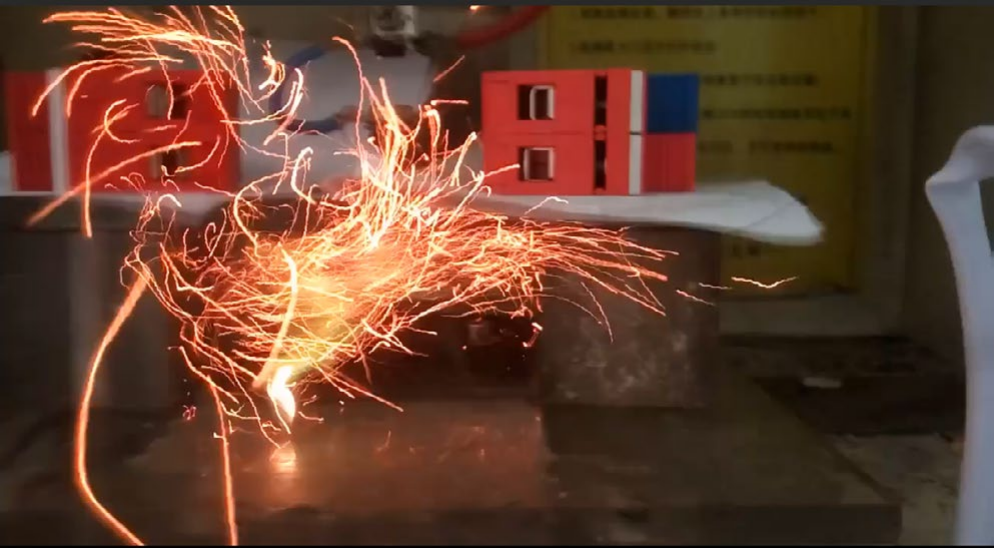
The ignition process of lightning strikes. The camera manufacturer is Samsung. The parameters of the *Larix gmelinii *(Ruprecht) Kuzeneva needle bed were as follows: *I*_p_ = 68.276 kA, *ρ* = 0.1698 g/cm^3^, *x* = 0%, and *h* = 3 mm. The waveform was 8/20 µs. The current direction was plate‐to‐electrode. Additionally, the videos of ignition and no ignition are shown in Video S1 and Video S2

The ignition phenomenon of the impulse current is shown in Figure 6.

At the beginning, the impulse current was discharged through the needle bed and produced intense light. After discharge, the needle bed was ignited, producing a large number of flames. As a result of the shock waves from the simulated discharge, the flames spilled out of the needle bed and cooled in the air. Some embers fell to the bench and kept burning. At the same time, a large amount of smoke was generated. After the end of combustion, the embers became black charcoals and white ashes.

### 3.2 Comparison of different conifer needle beds

The critical ignition results of the different conifer needle beds are shown in Table 3. We can determine that the needle bed of *L. gmelinii *(Ruprecht) Kuzeneva had the smallest average values of *I*_p_, $\int I^{2}dt$, and ∫UIdt for the critical ignition. The size of needles is one of the properties that has an effect on ignition probability. Smaller needles result in a larger contact area with the arc, and they can absorb more energy from the arc. The *L. gmelinii *(Ruprecht) Kuzeneva needles have the smallest diameter in this study; therefore, they can be ignited easily.

**Table 3:** Comparison of different conifer needle beds

	*I*_p_ (kA) of critical ignition	$\int I^{2}dt$ (A^2^s) of critical ignition	∫UIdt (J) of critical ignition	Breakdown voltage (kV)
Needle beds of *Pinus sylvestris *Linn. var. mongolica Litv.	101.19 ± 8.80	149,418 ± 26,272	6,650 ± 1,169	6.68 ± 0.58
Needle beds of *Pinus massoniana *Lamb.	94.76 ± 3.31	132,346 ± 9,426	6,055 ± 431	5.08 ± 0.61
Needle beds of *Pinus pumila *(Pall.) Regel	102.87 ± 7.29	154,918 ± 22,500	7,160 ± 1,040	6.90 ± 0.59
Needle beds of *Picea jezoensis *Carr. var. komarovii (V.Vassil.) Cheng et L.K.Fu	82.64 ± 6.78	100,021 ± 16,552	4,590 ± 760	6.85 ± 0.63
Needle beds of *Larix gmelinii *(Ruprecht) Kuzeneva	33.67 ± 3.32	16,603 ± 3,325	803 ± 161	7.52 ± 0.32

Note

The values are average and *SD*, respectively.

The breakdown voltage results of different conifer needle beds are shown in Table 3. The breakdown voltage has been used in many studies to measure the breakdown performance of materials (Liang, Chen, & Zhou, 2003; Lv et al., 2014). Based on the average breakdown voltage, the average breakdown voltage of the *P. massoniana* Lamb. needle bed is the smallest under these conditions. Similar to the results of Lv et al. (2014) and Zhou (2015), there is a moderate *SD* in the breakdown voltage of the substances.

### 3.3 Ignition under different impulse current waveforms

The ignition results of different impulse current waveforms are shown in Table 4.

**Table 4:** Critical ignition under different impulse current waveforms

	*I*_p_ (kA)	$\int I^{2}dt$ (A^2^s)	∫UIdt (J)
8/20 µs the current direction of plate‐to‐electrode	69.83 ± 3.92	74,943 ± 8,432	3,186 ± 358
8/20 µs the current direction of electrode‐to‐plate	67.11 ± 5.79	73,440 ± 13,087	3,454 ± 616
10/350 µs the current direction of plate‐to‐electrode	11.05 ± 0.52	37,400 ± 3,475	1,670 ± 226

Note

The values are average and *SD*, respectively.

Lightning energy ∫UIdt can be divided into Joule heat and mechanical energy. The energy that ignites the needles is the internal Joule heat and the heat transferred to needles from the arc. When the ignition energy required by the needles is the same, the average values of *I*_p_, $\int I^{2}dt$, and ∫UIdt under different current directions have few differences. The difference in the *I*_p_ values was only 3.9%. In addition, the average of total energy ∫UIdt input by the 8/20 µs waveform is larger than that of the 10/350 µs waveform, and the energy ratio for heating needles is less for the 8/20 µs waveform.

### 3.4 Prediction model of lightning ignition

**Figure 7:**
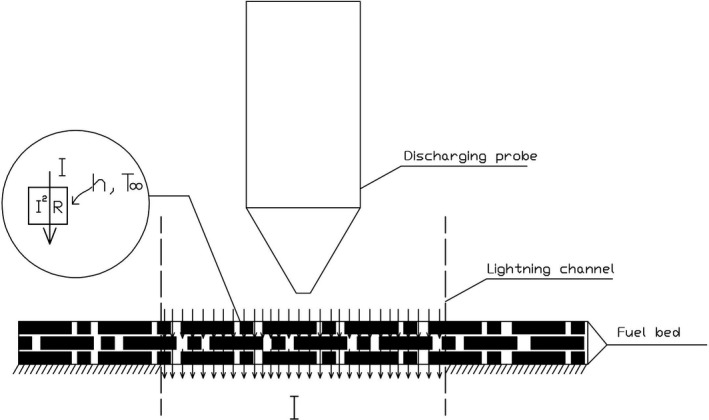
The ignition mechanism of the conifer needle bed during the lightning strike

Referring to the Anderson model, the ignition mechanism of conifer needles beds during lightning strikes can be simplified as shown in Figure 7. The needles are subjected to their own Joule heat and the heat transferred from the arc. Because the diameter of *L. gmelinii *(Ruprecht) Kuzeneva needles and the internal temperature gradient are very small, the needles can be regarded as hot thin solids with a uniform internal temperature.

A multiple log‐linear regression model was used to fit the relationships of *E*_c,impulse_ (J/mm) to *I*_p_, *ρ*, and *x*. It is shown in Equation 4:

(4) $$E_{\text{c,impulse}}=\int UIdt=0.2I_{p}^{2}\rho^{-0.05}{\left(x+1\right)}^{0.006}$$

**Figure 8:**
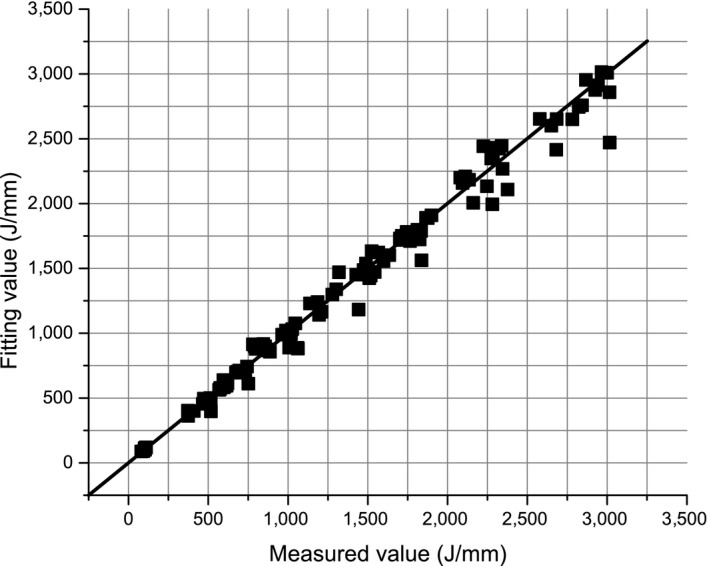
The measured values and fitted values of *E*_c,impulse_

The adj. *R*‐square is 0.965. The fitted values and measured values of *E*_c,impulse_ are shown in Figure 8. It can be seen that the multiple log‐linear regression model has a good effect on data fit. In addition, it can be seen from Equation 4 that the *E*_c,impulse_ values basically do not change with moisture content and bulk density.

The relationships of *φ* (%) to *I*_p_, *ρ*, and *x* were also fitted as a multiple log‐linear regression model. The results are shown in Equation 5. The adj. *R*‐square is 0.995. The prediction model of ignition (*φE*_c,impulse_ ≥ *E*_ig_ means ignition) is accurate.

(5) $$\phi =69564I_{p}^{-1.83}\rho^{1.12}{\left(x+1\right)}^{0.145}$$

The prediction model of LCC lightning ignition theory was obtained from Fuquay et al. (1979), and the *E*_c,LCC_ value of the LCC is shown in Equation 6:

(6) $$E_{\text{c,LCC}}=UIt=1\left(\text{V/mm}\right)\times 44.7t^{0.1787}\left(A\right)\times t\left(\text{ms}\right)=0.0447t^{1.1787}\left(\text{J/mm}\right)$$

*t*—the duration of LCC, ms.

Therefore, based on the conservation of energy, a lightning ignition prediction model of the full waveform that incorporates the heating effect of the impulse current can be derived (Equation 7):

(7) $$\phi E_{\text{c,impulse}}h+E_{\text{c,LCC}}h\geq \rho \pi r^{2}h\left\{c_{p}\left(T_{\text{ig}}-T_{0}\right)+x\left[c_{\text{pl}}\left(T_{s}-T_{0}\right)+h_{\mathrm{lg}}\right]\right\}$$

## 4 CONCLUSIONS AND DISCUSSIONS

In nature, lightning usually strikes trees because of their height and their electrical characteristics relative to the air (Defandorf, 1955). However, if we want to simulate the entire lightning process, we need very high‐voltage discharge equipment, which is difficult to achieve under the current conditions. This paper only takes into account the discharge process of needle beds on the forest floor. In nature, if the needle bed is far from the tree (e.g., the needles are affected by the wind and accumulate away from the tree on the edge of the forest), or the tree is very small and dry and the moisture content of the needle bed is high, the lightning may strike the needle bed directly. In another case, after man‐made logging or after death and collapse, trees lose their ability to attract lightning. The lightning may strike the needle bed on the forest floor directly. Coincidentally, the edge of forest land and logging sites are areas where lightning fires frequently occur (Shu et al., 2003). In addition, many studies (Latham & Schlieter, 1989; Zhu et al., 2012) also studied the fuel bed separately to reduce the complexity of the experiment.

We focused on the impulse current of lightning because there is already a long history of the long continuing current research. The experimental results show that the impulse current also has a considerable heating effect. In addition, the impulse current causes mechanical damage. Different sample types and current waveforms influence the ignition characteristics. The total energy of the lightning needed for ignition of the needle bed of *L. gmelinii *(Ruprecht) Kuzeneva is the smallest.

Via the artificial discharge experiments, a large number of quantitative experiments can be carried out. The mechanism of lightning fires can then be further explored. We established an ignition prediction model based on the impulse current. As the current increases, the total energy of the impulse current increases, which is similar to Joule's law. However, the increase in the current will decrease the heating efficiency. When applying the prediction model, it is necessary to know the bulk density, the moisture content, and the value of the impulse current in advance. Then, the prediction model can be used to determine whether the artificial impulse current can ignite the needle bed.

Our research provides a reference for experimental methods of producing lightning fires and increases our understanding of lightning ignition. The trends we present here suggest that lightning ignition depends on the electrical and physical characteristics of lightning and conifer needles, but additional sampling is needed to test for statistically significant differences among species. In the future work, we will compare the results from the lightning ignition prediction model based on the impulse current generator with lightning fire records to further verify the accuracy of the prediction model.

## CONFLICT OF INTEREST

The authors declare no conflicts of interest.

## AUTHOR CONTRIBUTIONS

Junwei Feng written the article, involved in experiment, analyzed the data, and designed the model; Hao Shen revised the article and analyzed the data; Dong Liang collected the data and designed the experiment.

## Supporting information

The Figure S1, Table S1, Video S1 and Video S2 are deposited in the Dryad data repository: https://doi.org/10.5061/dryad.x69p8czdf

## Data Availability

Our data is deposited to the Dryad data repository: https://doi.org/10.5061/dryad.x69p8czdf.
