# Supplementary figures and images for: Investigation of lightning ignition characteristics based on an impulse current generator
Source: Ecol Evol. 2019 Dec 2;9(24):14234–43. doi: 10.1002/ece3.5855 (PMC6953689; doi:10.1002/ece3.5855)

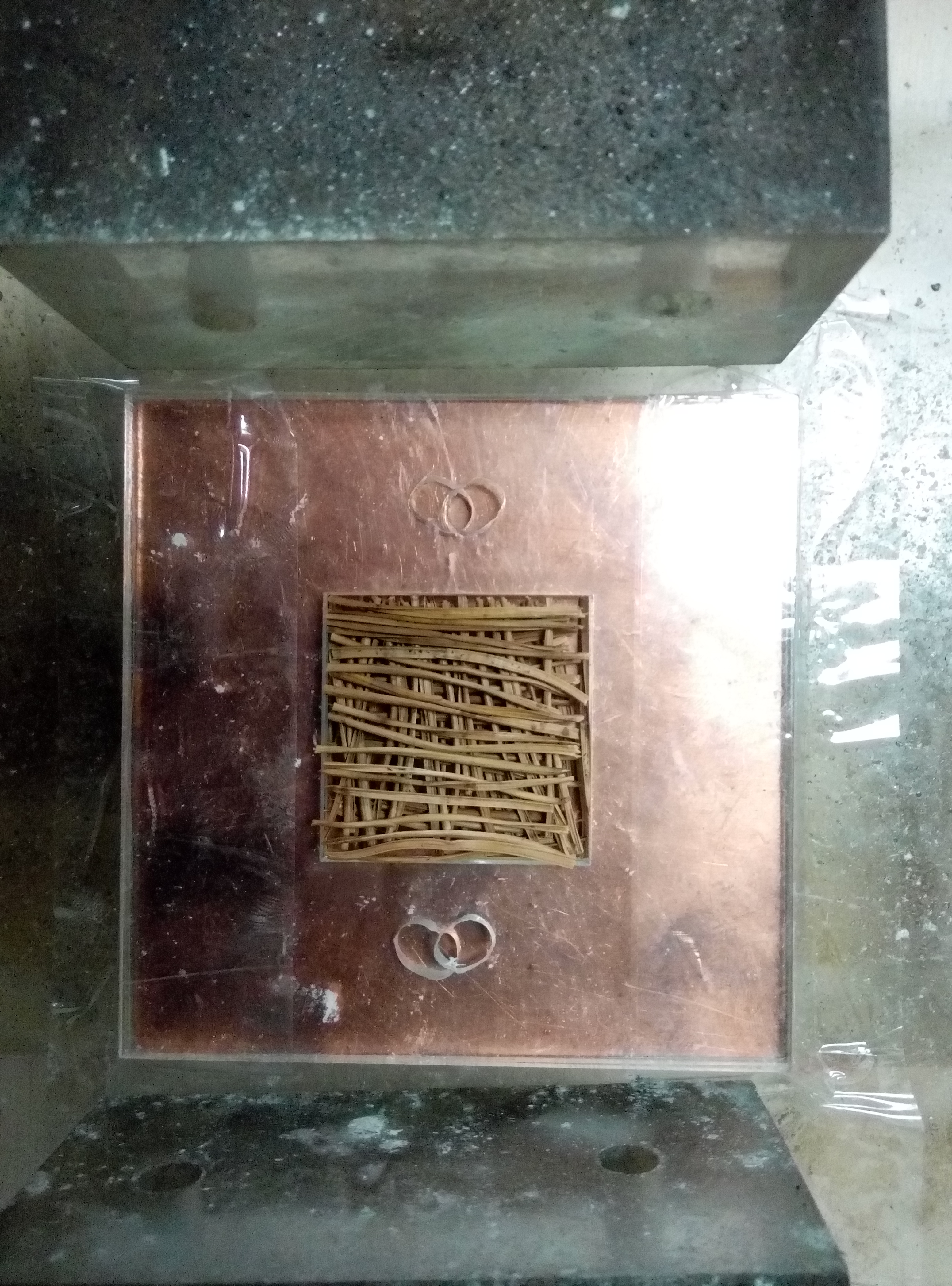

Supplement: Supplementary file 1 — The Figure S1, Table S1, Video S1 and Video S2 are deposited in the Dryad data repository: https://doi.org/10.5061/dryad.x69p8czdf [file ECE3-9-14234-s001.jpg]
