# Supplementary material for: Investigation of lightning ignition characteristics based on an impulse current generator
Source: Ecol Evol. 2019 Dec 2;9(24):14234–43. doi: 10.1002/ece3.5855 (PMC6953689; doi:10.1002/ece3.5855)
Supplement: Supplementary file 2 [file ECE3-9-14234-s002.docx]

**Supplementary Table 1. The technical parameters of the impulse current generator.**

|  | technical parameter description |
| --- | --- |
| DC charging power supply | rated capacity: 15kVA  rated input voltage: AC220 V  rated input current: 63 A  rated output voltage: DC60 kV  rated output current: 250 mA |
| 8/20 µs circuit | total capacitance: 32 µF  total resistance of wave modulation: 0.23 Ω  total inductance of wave modulation: 2 µH |
| 10/350 µs circuit | total capacitance: 512 µF  total resistance of wave modulation: 0.95 Ω  total inductance of wave modulation: 3 µH |
